# Endothelial Dysfunction and Hyperhomocysteinemia-Linked Cerebral Small Vessel Disease: Underlying Mechanisms and Treatment Timing

**DOI:** 10.3389/fneur.2021.736309

**Published:** 2021-11-24

**Authors:** Shuang Li, Guangjian Li, Xia Luo, Yan Huang, Lan Wen, Jinglun Li

**Affiliations:** ^1^Department of Neurology, The Affiliated Hospital of Southwest Medical University, Luzhou, China; ^2^Laboratory of Neurological Diseases and Brain Function, The Affiliated Hospital of Southwest Medical University, Luzhou, China; ^3^Department of Neurology, Southwest Hospital, Third Military Medical University (Army Medical University), Chongqing, China; ^4^Department of Neurosurgery, West China Hospital, Sichuan University, Chengdu, China

**Keywords:** cerebral small vessel disease, homocysteine, hyperhomocysteinemia, endothelial dysfunction, homocysteine-lowering therapy

## Abstract

Cerebral small vessel disease (cSVD)—a common cause of stroke and vascular dementia—is a group of clinical syndromes that affects the brain's small vessels, including arterioles, capillaries, and venules. Its pathogenesis is not fully understood, and effective treatments are limited. Increasing evidence indicates that an elevated total serum homocysteine level is directly and indirectly associated with cSVD, and endothelial dysfunction plays an active role in this association. Hyperhomocysteinemia affects endothelial function through oxidative stress, inflammatory pathways, and epigenetic alterations at an early stage, even before the onset of small vessel injuries and the disease. Therefore, hyperhomocysteinemia is potentially an important therapeutic target for cSVD. However, decreasing the homocysteine level is not sufficiently effective, possibly due to delayed treatment, which underlying reason remains unclear. In this review, we examined endothelial dysfunction to understand the close relationship between hyperhomocysteinemia and cSVD and identify the optimal timing for the therapy.

## Introduction

*Cerebral small vessel disease* (cSVD) refers to a series of clinical, imaging, and pathological syndromes caused by various etiological factors affecting small arteries, capillaries, and venules in the brain. As an age-dependent disease, the cSVD incidence is predicted to increase with the aging population (1). Several factors can contribute to the occurrence of cSVD, such as age, hypertension, diabetes, hyperlipidemia, small artery sclerosis, cerebrovascular amyloid, gene mutations, immune-mediated vasculitis, and certain infections (2); arteriolar sclerosis is the most common factor in older individuals (3, 4).

Globally, cSVD causes up to 45% of dementia and accounts for approximately 20% of all strokes. Twenty-five percent of these stroke cases are of the ischemic (or lacunar) type, of which in about 20% patients left disabled (3), putting an enormous burden on the society and families of these patients. Evidence regarding the efficacy of treatment with antiplatelets, anticoagulants, lipid-regulating drugs, and other methods in cSVD is lacking (5–7). Therefore, early detection and intervention of the risk factors is currently a beneficial strategy (8). The characteristic magnetic resonance imaging (MRI) markers are white matter hyperintensities (WMH), lacunes of presumed vascular origin, cerebral microbleeds (CMBs), enlarged perivascular spaces (EPVS), and cerebral atrophy (9, 10). Apart from imaging markers, sensitive, specific, and simple serum markers, such as homocysteine (Hcy), intercellular adhesion molecule-1 (ICAM-1), C-reactive protein (CRP), and interleukin-6 (IL-6) are also valuable in diagnosing cSVD (11).

Homocysteine (Hcy) is also an age-dependent indicator, and an elevated serum total Hcy (tHcy) level is associated with cardiovascular diseases, atherosclerosis, cerebrovascular diseases, and neurodegenerative disorders (12). Therefore, Hcy-lowering treatment is gradually becoming a therapeutic target for these diseases. However, contradictory results have been reported in cSVD. Several trials reported that primary prevention with Hcy-lowering therapy could reduce the incidence of cerebrovascular diseases (13–15). Unfortunately, a few trials using vitamin B supplementation to reduce Hcy levels failed to demonstrate the benefit of reducing stroke and cognitive impairment events (16–18). Understanding the mechanism behind Hcy-lowering therapy could help elucidate these findings. Endothelial dysfunction (ED) is a core mechanism of hyperhomocysteinemia (HHcy) affecting cSVD. Reducing Hcy levels alleviates ED, and early intervention of ED could reverse cSVD progression. Therefore, it is critical to identify the correct timing of lowering serum Hcy levels for treating ED and improving cSVD patient's prognosis.

## Hyperhomocysteinemia

### Homocysteine Metabolism and HHcy

**Figure 1:**
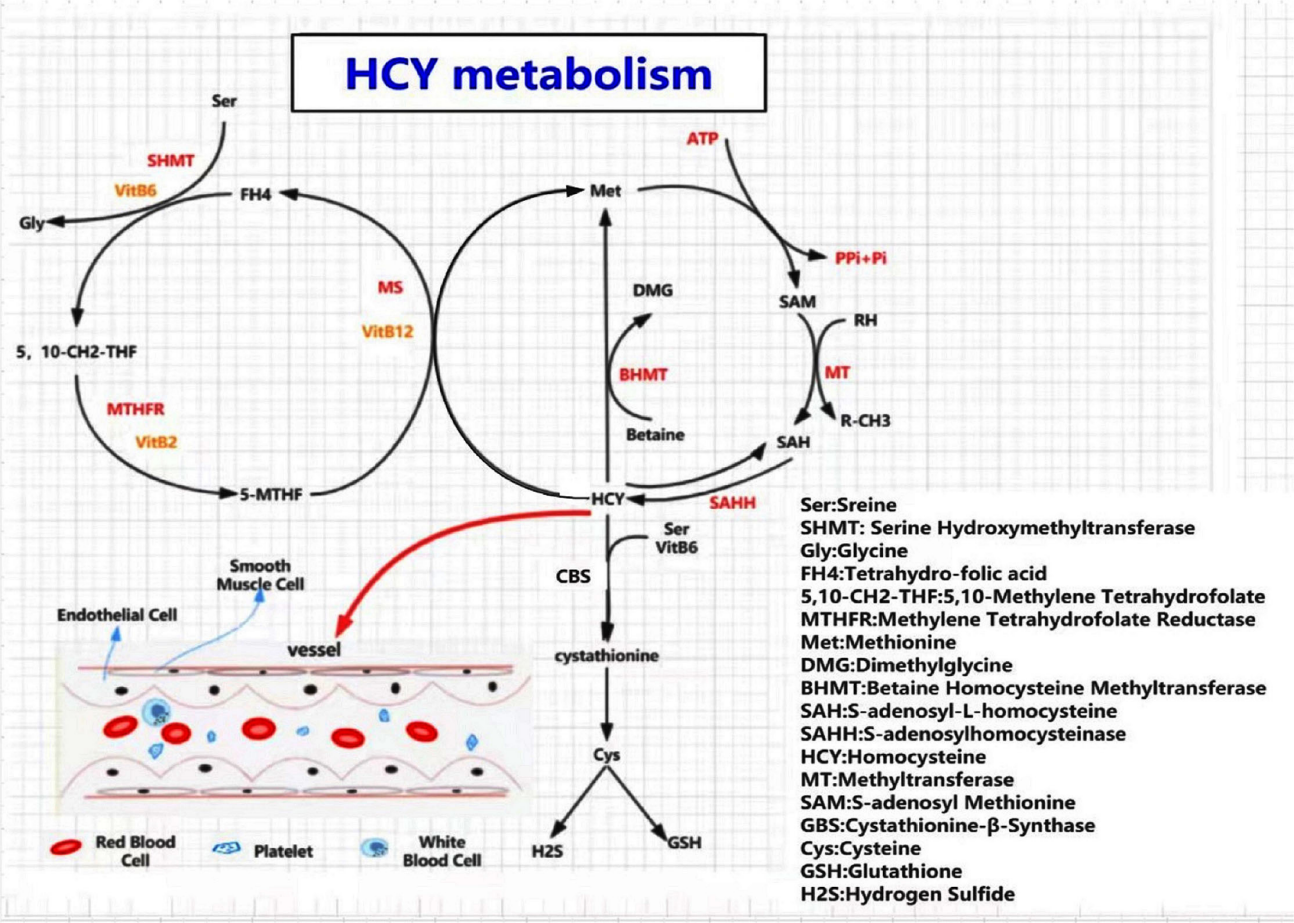
A diagram of homocysteine (Hcy) and methionine metabolism. Three pathways involved in Hcy meatabolism, including remethylation pathway, transsulfuration pathway, and a small amount of Hcy released directly into the extracellular fluid. When a deficiency of MTHFR, CBS, MS, or B-vitamins occurs, an excess of homocysteine enters the blood, causing HHcy, resulting in damage to the corresponding target organs. SAM is the only donor of methyl in all of the metabolic pathways. HHcy leads to hypomethylation of biomolecules, such as proteins, lipids, and nucleic acids, which contributes to metabolic abnormalities and promotes disease occurrence.

Hcy is an intermediate metabolite in the methionine cycle, and the only Hcy sources are methionine-containing foods, such as eggs, sesame seeds, nuts, and meat. Plasma Hcy concentrations increase with age and are higher in men than in women (12, 19, 20). Hcy is primarily involved in methyl transfer (or one-carbon metabolism) and can undergo three metabolic pathways (21–23) (Figure 1): (a) *Remethylation:* Hcy is remethylated with the participation of 5-methyltetrahydrofolate and vitamin B12 (cobalamin) and catalyzed by methionine synthase (MS) to form methionine. Alternatively, betaine provides methyl to produce methionine catalyzed by betaine-homocysteine methyltransferase (BHMT); (b) *Transsulfuration Pathway:* Hcy is formed as cysteine is catalyzed by cystathionine beta-synthase (CBS) and its coenzyme, vitamin B6; (c) a small amount of Hcy is released directly into the extracellular fluid to perform physiological functions.

Vitamins B2, B6, B9 (folic acid), and B12 are indispensable coenzymes in the metabolism of the amino acid Hcy, and their deficiency could disturb the aforementioned pathways, leading to Hcy accumulation and elevated levels of Hcy in the plasma that triggers HHcy (24).

*HHcy* is defined by the plasma Hcy level of more than 15 μmol/L, and can be categorized into mild (Hcy = 15–30 μmol/L), moderate (Hcy = 30–100 μmol/L), and severe (Hcy >100 μmol/L) (24). HHcy can be classified as primary or secondary. Primary HHcy is mainly caused by mutations in the *MTHFR* (C677T or A1298C) and *MTRR* (A2756G) genes (25, 26). A higher *MTHFR* mutation rate is observed in the Chinese population than the others (27). The frequency of C677T mutation varies between 23.2 and 45.2% in the Chinese population (25), contributing to higher plasma-Hcy levels in Chinese than in Americans and Koreans (28–30). Secondary HHcy is mainly caused by impaired Hcy metabolism due to the deficiency of vitamin B2, B6, B9 (folic acid), or B12, renal failure, hypothyroidism, tumors, certain drugs, among several other causes (31). Dietary overdose may also lead to HHcy.

### HHcy and Disease Condition

As a characteristic feature of aging, HHcy is causally linked to the development of age-associated disorders, such as ischemic heart diseases, atherosclerosis, cerebrovascular diseases, neurodegeneration, glomerular filtration rate decline, bone fractures, and venous thrombosis (12, 32). In particular, the focus has been on cardiocerebrovascular events. Several studies have shown that interventions to reduce tHcy, such as B-vitamin supplementation alone or in combination, had no effect on myocardial infarction and death from any cause or adverse event; however, statistical differences were observed in the stroke group (33, 34). These results suggested that the nervous system may be more sensitive to the toxic effects of HHcy than the cardiovascular system. Similarly, a study on the secondary prevention of stroke found that the use of B-vitamin supplements to lower Hcy significantly reduced stroke events and recurrence (13–15). Regardless of a history of acute ischemic stroke, patients with higher plasma-Hcy concentrations are more likely to develop cognitive impairments and depression than patients with normal levels (35, 36). Consequently, HHcy is a significant risk factor for nervous system disorders, especially cerebrovascular diseases.

### HHcy Damages Brain Small Vessel Through Affecting Endothelial Function

There is an abundance of evidence that HHcy can damage brain small vessel, which increases the incidence of cSVD. The mechanisms include inflammation, atherosclerotic plaque formation, endothelial dysfunction, smooth muscle cell proliferation and oxidative stress response (37, 38). Among these pathological injury mechanisms, endothelial dysfunction plays a major role and determines the occurrence, development, and prognosis of cSVD (39). Hcy accumulation can interfere with the normal functioning of endothelial cells (ECs) through many pathological pathways (40–50). These include (a) oxidative stress resulting from the uncoupling of nitric oxide (NO) synthase, upregulation of the oxidation system, and decline of the antioxidant system; (b) competitive inhibition of NO synthase due to accumulation of asymmetric dimethylarginine (AMDA), resulting in decreased production of NO; (c) reduced hydrogen sulfide production, leading to abnormal vascular relaxation; (d) endoplasmic reticulum stress and unfolded protein response with eventual EC apoptosis; (e) chronic inflammation and prothrombotic conditions; and (f) epigenetic alterations, including hypomethylation and protein N-homocysteinylation. These mechanisms result in endothelial dysfunction and apoptosis, broken intercellular tight connections, and chronic hypoperfusion, which could be conducive to the formation and progression of cSVD.

## Endothelial Dysfunction and cSVD

### ED' Relevance in cSVD

Pathological changes in the small vessels vary during different periods of the cSVD process, including ED, blood-brain-barrier (BBB) injury, inflammatory response, smooth muscle proliferation, and small vessel sclerosis (3). Although different pathological changes play different roles in cSVD, they overlap and affect each other. ED triggers the first step of small blood vessel damage and plays a crucial role during the entire disease development process (39, 51). The significance of ED in cSVD has been well-established by population studies. A case-control study showed a worsening cerebral vasoreactivity in symptomatic lacunar stroke patients compared to that in matched controls, while cerebrovascular reactivity was used as a marker of endothelial function, indicating that ED severity in the cerebral artery may be a predisposing factor of symptomatic lacunar stroke in cSVD patients (51). Abnormal endothelial secretion of soluble ICAM-1, promotes the adhesion of leucocytes, which causes small vessels to occlude, and leads to incidence of silent brain infarctions and periventricular white matter lesions, implying ED in relation to progression of ischemic cerebral small vessel disease in type 2 diabetes (52). Markers of endothelial activation and damage, such as ICAM-1, thrombomodulin (TM), tissue factor (TF), and tissue factor pathway inhibitor (TFPI), were associated with the number of lacunes and severity of leukoaraiosis in the cSVD group in a prospective series of patients suffering from a lacunar strokes (53).

### How ED Affects cSVD

ECs in the brain maintain BBB's integrity and regulate transmembrane transport functions. In addition, ECs are functionally active in controlling normal physiological processes, such as blood clotting, matching blood flow to neural activity, inflammatory reaction, and angiogenesis (54). When ischemic attacks, immune inflammation, or oxidative stress damage the endothelium, various abnormally released molecules (e.g., vasomotor factors, adhesion molecules, inflammatory cytokines, factors involved in the coagulation process, and enzymes) from ECs lead to impaired endothelium-dependent vasodilation, BBB destruction, and pre-thrombotic state, and exacerbates cSVD progression (11).

**Figure 2:**
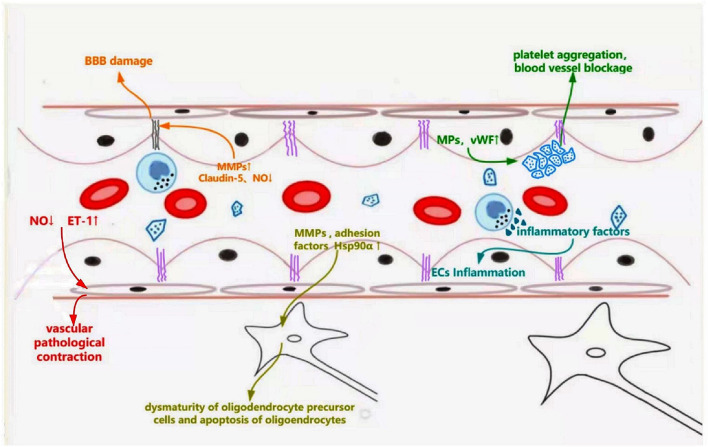
Schematic representation of dysfunctional ECs linking to specific features of cerebral small vessel disease. Dysfunctional ECs induce abnormal secretion of NO and endothelin-1, which leads pathological vascular contraction. Increased secretion of MMPs and decreased production of claudin-5 destroy the tight connections, leading to blood-brain barrier leakage. Abnormally released MMPs, adhesion factors (ICAM-1, VCAM-1), and Hsp90α result in dysmaturity of oligodendrocyte precursor cells and apoptosis of oligodendrocytes, which affects the myelination. Endothelial microparticles and vWF are released from the damaged ECs, causing platelet aggregation and inflammatory response.

ED is the first step in cSVD pathogenesis. It is associated with leukoaraiosis, lacuna infarctions, and microbleeds through multiple underlying mechanisms (Figure 2). Firstly, the imbalance of vasomotor factors leads to pathological vasoconstriction and blood flow reduction, eventually leading to ischemia in the corresponding regions. Endothelially-derived NO, a key factor of vasodilation, is produced by L-arginine in a reaction catalyzed by endothelial-type NO synthase (eNOS), released into smooth muscle cells, wherein it activates guanylate cyclase to relax the vessel mediated by the cGMP pathway (55). In eNOS-deficient ED mice, NO reduction leads to pathological vasoconstriction and cerebral hypoperfusion, which exhibits similar MRI findings to those of cSVD, such as microhemorrhage, microinfarction, and white matter changes (56). In a cSVD rat model, ED, subsequent NO reduction, and BBB injury were associated with WMH burden (57). In addition, dysfunctional ECs can increase the secretion of endothelin-1, a vasoconstrictor, resulting in the pathological process of vasoconstriction (39).

Secondly, ECs and their tight junction (TJ) protein complexes are required to form a complete BBB. TJ protein complexes between ECs maintain BBB integrity and control the passage of cells and molecules. Damaged ECs and broken tight junctions can decrease BBB's integrity and increase its penetrability, leading to inflammation and edema of the surrounding tissue; corresponding signal changes can be observed on an MRI (58). The destruction of TJs caused by ECs dysfunction mainly results from the abnormal secretion of NO, matrix metalloproteinases (MMPs), and claudin proteins, inhibiting the modification of TJ proteins via nitrosylation or nitrosation, promoting TJ loss and accelerating BBB leakage (59). MMP-2 and MMP-9 also show increased expression, leading to decreased TJ proteins and transmembrane protein, claudin-5, which increases BBB's permeability (60). In addition, ED reduces the release of claudin-5, which adversely affects the tight connections between ECs and causes BBB injuries, which can impair brain function (61).

Thirdly, the survival and maturation of oligodendrocytes could be affected by ED and eventually interfere with white matter myelination. In an ATP11B-knockout rat model of cSVD, dysfunctional vascular endothelium secretes MMP chaperone protein, heat shock protein 90α (HSP90α), which leads to the maturation of oligodendrocyte precursor cells, contributing to white matter changes (57). Excessive MMPs, especially MMP-3 and MMP-9, secreted from dysfunctional ECs into the brain tissue cause and accelerate myelin breakdown (62). Moreover, dysfunctional ECs secrete increased CRP, IL-6, ICAM1, and e-selectin, leading to oligodendrocyte damage, and even apoptosis (4).

Finally, ED can participate in cSVD pathogenesis in multiple ways. Elevated plasma adhesion factors (ICAM-1 and vascular cell adhesion molecule-1 [VCAM-1]) and endothelial microparticles result from ED activating circulating inflammatory cells and causing vascular inflammation (63–65). Increased release of von Willebrand factor (vWF) caused by ED, which acts as a bridge to connect the process of platelets and inflammation, could lead to small vessel occlusion and further stimulate inflammation (66). These changes affect the structure and function of small vessels, contributing to the occurrence of cSVD and participating in its progression.

### ED and Disease Reversal in the Early cSVD Stage

ED could be reversed through therapeutic interventions targeting the risk factors and inhibiting endothelial oxidative stress and inflammation. Previously, relieving eNOS uncoupling has been shown to reverse ED (54). This theory is also suitable for cSVD. Drug treatment to stabilize ED reverses the endothelial and oligodendroglial pathologies. Improved endothelial function can reverse ED itself, WMH, and oligodendroglial changes in the early disease state (57). Evidence suggests that in the early stage of cSVD, punctate lesions and a small number of white matter lesions caused by impaired interstitial fluid circulation may be quiescent and reversible. In contrast, fused and massive white matter lesions caused by demyelination and chronic hypoperfusion at later stages generally appear progressive and irreversible (67, 68). Thus, reversal of ED is a crucial therapeutic mechanism of cSVD, and the time window is notable and worth considering.

## HHcy and cSVD

Several population-based experiments confirmed that elevated baseline Hcy levels are positively associated with cSVD incidence and the subsequent severity and disease progression, independent of vascular risk factors and severity of the atherosclerotic disease. The relevance of the presence and number of lacunar infarcts, WMH volume, and progression to high baseline Hcy levels (32, 69–71) provides a more intuitive view of the correlation between HHcy and cSVD. In patients with dementia caused by small vessel lesions, higher tHcy levels were used to predict WMH progression, and the level was associated with deep CMBs and lacunes (32). In cSVD-related stroke, serum Hcy levels were significantly higher in patients with large WMH volumes than those with small WMH volumes (69, 70). The association between Hcy and WMH burden suggested that the degree of ED may be greater in patients with increased WMH volume and may, in part, explain that ED acts as a bridge between HHcy and cSVD (72). Similar results were obtained in recent observational studies, where WMH volume and EPVS number were positively correlated with plasma Hcy concentrations. EPVS may be specifically associated with BBB leakage led by injured ECs and is considered as an important marker of BBB disruption (68, 73, 74).

### ED in Hcy-Linked cSVD

**Figure 3:**
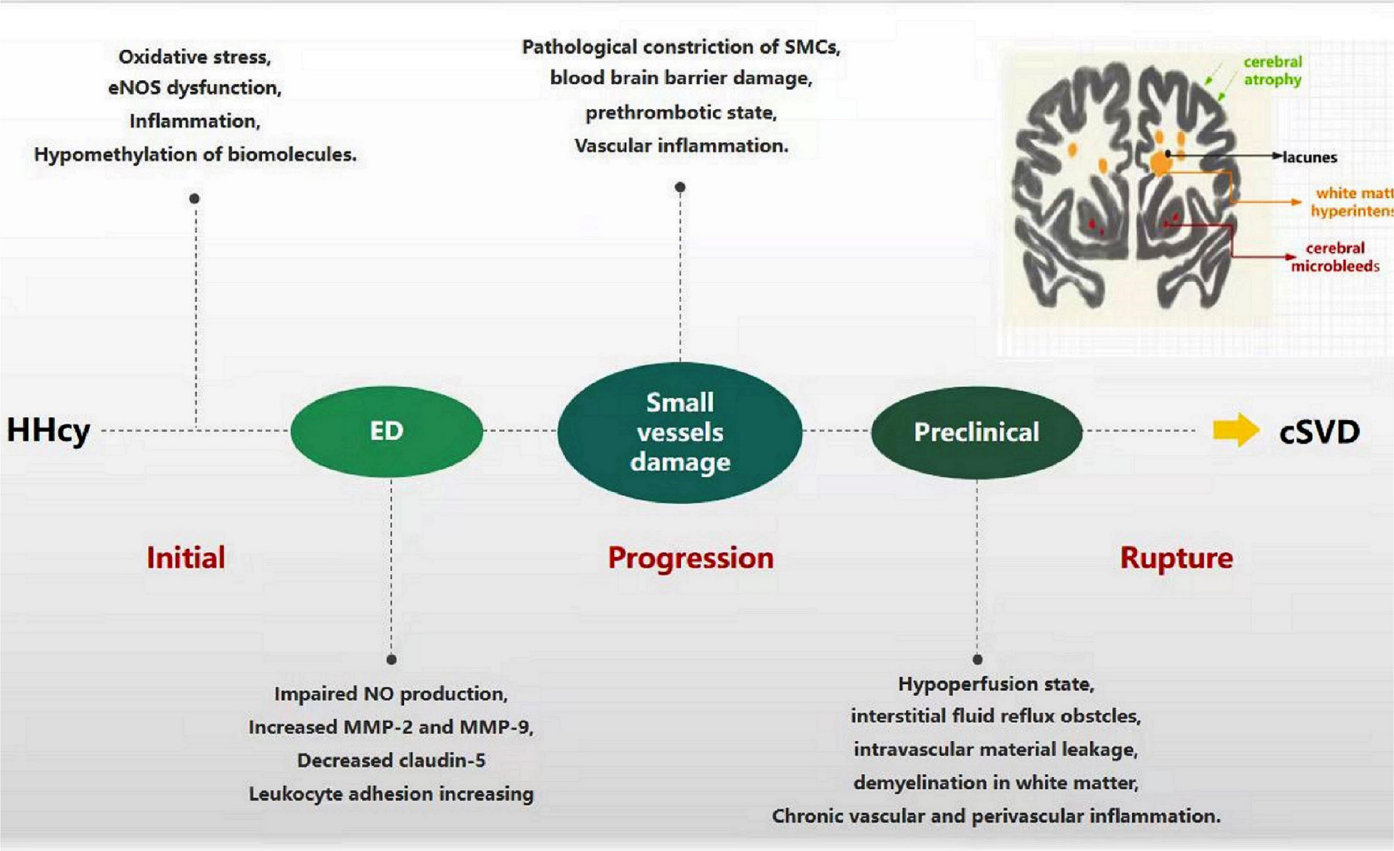
Progression of HHcy-induced cSVD through ED. In the beginning, HHcy induces endothelial cell injury through oxidative stress, eNOS dysfunction, inflammation, and hypomethylation of biomolecules. Dysfunctional endothelia release the molecules abnormally, such as impaired NO production, increased MMPs and adhesion factors, decreased claudin-5. The process facilitates the damage of small vessels, including pathological vasoconstriction, SMCs injury, blood-brain barrier damage, pre-thrombotic state and vascular inflammation. Along with these pathological changes progression, hypoperfusion, intravascular material leakage, demyelination, chronic vascular and perivascular inflammation appear successively, which eventually develop into cSVD.

As mentioned earlier, ED is a major mechanism of HHcy-linked cSVD, which mainly manifests as NO reduction, TJ breakage, BBB leakage, platelet aggregation, and pre-thrombotic state (Figure 3). The decrease in NO production and bioavailability predominates in Hcy-induced endothelial injury models, which exhibits vasoconstriction, vascular smooth muscle proliferation, and small arteriosclerosis; inadequate perfusion eventually develops cerebral white matter damage, acute small infarctions, and hemorrhages (44, 45). In addition, HHcy damages ECs and induces abnormal secretion of MMPs and transmembrane proteins, disrupting endothelial junctions, leading to BBB leakage, providing an important pathological basis for WMH, EPVS, lacunes, and CMB (75, 76). Furthermore, HHcy can also induce a pre-thrombotic state by modifying fibrinogen or enhancing platelet activation and coagulation and weakening fibrinolysis, which is prone to thrombosis and leads to ischemic stroke (42, 77). Naturally, Hcy-lowering therapy acts by ameliorating ED. AVE3085, a specific eNOS-targeting enhancer, prevents Hcy-induced ED by increasing NO production and reversing eNOS reduction in human ECs. The protective effects of AVE3085 translate into improved endothelium-dependent relaxation in the aorta of spontaneously hypertensive rats and significantly improved endothelium-dependent vasorelaxation in the Hcy-exposed human internal mammary artery (54). However, these studies indicate that ameliorating ED could reverse disease progression, which seems to be working just at early stage.

### Hcy-Lowering Therapy in cSVD

In HHcy patients, several trials, reviews, and meta-analyses evaluating primary prevention with treatment, have shown that Hcy-lowering therapy with B-vitamin supplementation reduces the incidence of cerebrovascular diseases (13–15), in contrast to studies that have shown no such association (16–18). Thus, the main question might be at what stage of the disease can cSVD lesions be reversed or stopped from progressing using proper Hcy-lowering treatment. Some studies did not observe positive results due to malabsorption and other individual factors. Patients with chronic kidney disease and those on antiplatelet therapy had reduced effects of B-vitamin supplementation on Hcy-associated cerebrovascular events (78). Older patients with gastrointestinal diseases failed to reach therapeutic concentrations of B-vitamins due to malabsorption and Hcy-lowering therapy may not be effective for hereditary cSVD (79). Moreover, delayed treatment may be a significant cause of a poor response to treatment. Nam et al. showed that if an ischemic stroke occurs in cSVD patients, disease progression might not be reversed by treatments meant to lower the tHcy levels, which suggest that patients with early or preclinical cSVD are more likely to benefit from Hcy-lowering therapy, and the curative effect was closely related to the timing of the treatment (68). In the stroke prevention trial, patients with low baseline Hcy levels had better outcomes than those with high levels (80). It indicates that Hcy-lowering treatment is similar to statin therapy for lowering lipid levels, and the underlying disease mechanisms are independent of the original baseline Hcy level. Considering that Hcy levels are associated with disease severity, it is also possible that patients with lower levels are more likely to be at an early stage of the disease and are more likely to benefit from treatment.

## Discussion

Both cSVD and HHcy are age-dependent diseases, and as a risk factor of cSVD, HHcy contributes to its onset and progression. Nonetheless, the use of Hcy-lowering therapy is controversial. Therefore, it is unclear whether Hcy can be considered as an early indicator of cSVD and whether reducing Hcy levels is necessary for the treatment. Considering the prevalence of HHcy in older individuals and their vulnerability to cSVD, monitoring of tHcy levels is recommended. In consideration of the limited treatment time window, active measures should be taken sooner rather than later. ECs were injured when first exposed to elevated tHcy conditions; once Hcy is elevated, damage to the endothelium begins, which precedes cerebral small vessel injury years before the onset of cSVD. As mentioned above, ameliorating ED could reverse the progression of the disease in its early stage, which is the primary purpose of Hcy-lowering therapy. Since effective therapy relies on early intervention, immediate steps should be taken upon detection of HHcy. Currently, most pathological states are described as early or later stages; therefore, the critical point of the irreversible stage remains unclear. Further studies are needed to evaluate the relationship between different manifestations of endothelial injury and determine the timeline of brain damage in patients with cSVD.

## Author Contributions

All authors listed have made a substantial, direct and intellectual contribution to the work, and got involved in the process of preparation, correction, and modification of the manuscript. All of them approved it for publication.

## Funding

LW was a post-doctoral fellow from the West China Hospital of Sichuan University and was supported by Postdoctoral Research Found of China (2021M692285).

## Conflict of Interest

The authors declare that the research was conducted in the absence of any commercial or financial relationships that could be construed as a potential conflict of interest.

## Publisher's Note

All claims expressed in this article are solely those of the authors and do not necessarily represent those of their affiliated organizations, or those of the publisher, the editors and the reviewers. Any product that may be evaluated in this article, or claim that may be made by its manufacturer, is not guaranteed or endorsed by the publisher.
